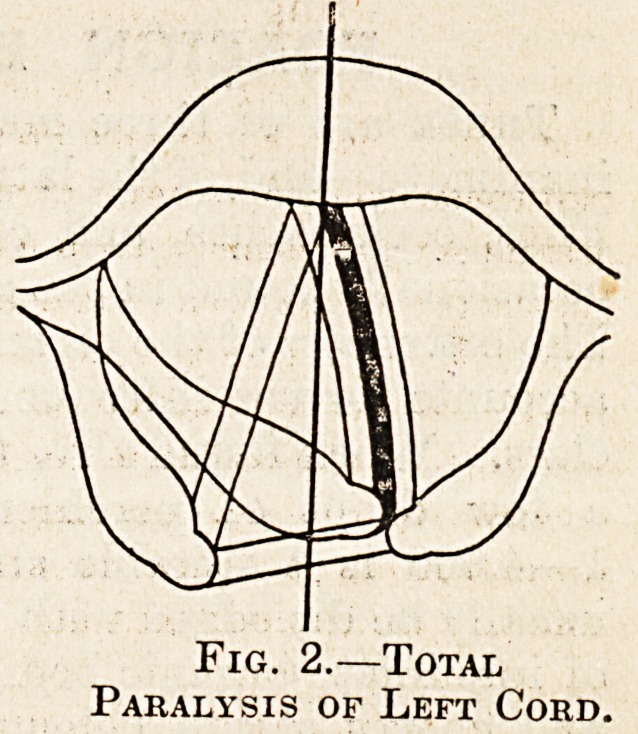# Laryngeal Paralyses and Their Diagnostic Value

**Published:** 1907-06-29

**Authors:** 


### Laryngology and Rhinology.

### LARYNGEAL PARALYSES AND THEIR DIAGNOSTIC VALUE.

Paralysis of the vocal cords is an early manifestation of several important and insidious diseases; consequently the recognition of these paralyses, and the knowledge of their causes, are aids to diagnosis which the practitioner cannot afford to neglect. It is necessary, too, to remember that the voice may remain quite normal in unilateral abductor paralysis, which of all these palsies is the form most frequently found, and therefore that diagnosis is impossible without laryngoscopic examination. We are speaking only of organic paralysis, and not of "functional aphonia," or the adductor forms of palsy.

One pair of muscles, the crico-arytenoidei postici, act as abductors of the cords; each rotates the vocal process of its arytenoid outwards, and also causes a gliding movement of the whole cartilage outwards and backwards. Of adductors there are two pairs and one unpaired muscle; the crico-arytenoidei laterales rotate the vocal processes inwards, the arytenoideus draws the cartilages together, and the thyro-arytenoidei make straight and tense the edges of the vocal cords. These muscles are endowed with very unequal powers of resistance, and in any gradually progressive lesion of the nerve-path they become paralysed in a certain definite order, the enunciation of which is known as Semon's law. The abductor muscle fails first, and may remain for an indefinite time the only one affected; then the thyro-arytenoideus, or "internal tensor," of the cord becomes paralysed, and finally the crico-arytenoideus lateralis.

The appearances thus produced are characteristic and easily recognised. When abductor paralysis has occurred the affected cord lies in the middle line; during phonation the healthy cord adducts to meet it, and the larynx appears normal, but on inspiration the sound arytenoid is drawn outwards and backwards, making the paralysed cord, which remains in the middle line, appear the shorter. There is no alteration in the voice, but, as the maximum available aperture is reduced by half, there will be some dyspnœa on active exertion, though there is no obstruction to quiet breathing except in children. When the thyro-arytenoideus muscle fails, the edge of the cord is concave on phonation and the voice becomes gradually hoarse. Finally, when total recurrent paralysis has resulted, the cord assumes the "cadaveric" position between the middle line and the normal position of rest; during inspiration the sound cord lies abducted and behind its fellow, but on phonation it crosses the middle line and pushes the paralysed cartilage aside. The paralysed arytenoid sometimes drops forwards, exposing its broad posterior surface to view, an appearance which may easily be mistaken by the inexperienced for an infiltration or a tumour. Even now the voice is not lost, but is weak and readily tired; it is of a peculiar low-pitched hoarseness, quite different from the huskiness of catarrh, the raucous voice of syphilis, or the weak aphonic voice of tuberculous laryngitis. In cases of bilateral abductor paralysis both cords lie in the middle line; phonation is good, but there is marked inspiratory dyspnœa with exacerbations; when the adductors also fail the dyspnœa becomes less severe and the voice is completely aphonic.

The diagnosis of these conditions is usually quite easy with the laryngoscope, and in unilateral cases the appearance of one arytenoid lying asymmetrically behind the other is very suggestive. Confusion may be caused by obliquity of the image due to a crooked position of the mirror, but the movements of the cords are equal, and the difficulty vanishes on placing the mirror correctly or on introducing it with the other hand. In nervous people under examination the cords are sometimes imperfectly separated on inspiration, thus giving the appearance of double abductor paralysis; but if the patient be made to phonate for as long as possible the cords will abduct naturally during the involuntary gasp which follows. The arytenoid cartilage may be

Fig. 1.—Abductor Paralysis of Left Cord.

Fig. 2.—Total Paralysis of Left Cord.

fixed, usually near the cadaveric position, as a result of inflammation about the joint; there are usually to be seen scars, swelling, or deformity, but even when this is not the case the diagnosis can often be made by observing that the arytenoid remains quite motionless, and is not pushed away by its fellow on phonation.

The importance of these paralyses to the practitioner lies in a knowledge of their causation, for they are often the earliest manifestation of serious organic disease. The cords are controlled bilaterally from both cerebral hemispheres, and therefore a unilateral lesion above the bulbar nuclei will not cause paralysis; thus the larynx is unaffected in ordinary hemiplegia. The causal lesion may be situated (1) in the medulla, (2) at the base of the brain, (3) in the vagus, or (4) in the recurrent laryngeal nerve, but it must be confessed that it frequently remains unlocated. Paralyses of bulbar origin are often bilateral, and neighbouring nuclei are liable to be affected as well; thus paralysis of one cord and of the same side of the palate is an important symptom-complex, and a few cases have been recorded of paralysis of cord, palate, sterno-mastoid and trapezius from involvement of the bulbar and spinal accessory roots. Persistent frequency of the pulse, due to disease of the cardio-inhibitory centre, is an important sign of bulbar disease. Tabes dorsalis is such a frequent cause of cord-paralysis that it should be thought of in all cases, and it may be for years the only manifestation; abductor palsy is also common in general paralysis. Syphilitic disease at the base of the brain is a frequent cause, and here the ocular muscles are often also affected. In bulbar paralysis laryngeal palsy is the rule, but rarely appears until late in the disease. Of peripheral causes, neuritis is a more frequent factor than has been until lately generally recognised; it may be toxic, due usually to lead, rarely to alcohol or arsenic; or infective, chiefly from diphtheria, less often from typhoid, influenza, or other fevers; there is also a "primary" neuritis, analogous to Bell's facial palsy, where the affection is transient and the only cause appears to be "cold." Peripheral lesions are, however, more often mechanical, and usually affect the recurrent laryngeal nerve, and especially, owing to its peculiar course, that of the left side. The most frequent of these causes are, in order, (1) aneurysm, (2) enlarged glands, usually tuberculous, and (3) cancer of the œsophagus; rarer causes are mediastinal growths, pulmonary tubercle, cancer of the lung, pleurisy, goître, and massive pericardial effusion. The commonest peripheral causes of bilateral paralysis are œsophageal cancer and goître. Aneurysm is the most frequent of all causes, and the resulting cord-paralysis may be for long the only symptom, therefore a guarded prognosis should be given in all cases of paralysis of unknown origin. Tuberculosis is a fairly frequent cause of paralysis, either by pressure of diseased glands or by involvement of the nerve in an infiltration of the apex of the lung; in the latter case the right recurrent nerve is the most often affected, so that paralysis of the right cord should suggest a suspicion of phthisis. The association of laryngeal paralysis with a thyroid tumour, though suspicious, is not conclusive of malignancy, for an innocent goître, if increasing rapidly in size, may compress the nerve.

Enough has been said to show that paralysis of the vocal cords is an important aid to diagnosis in many serious, and often obscure, organic diseases.

## Figures and Tables

**Fig. 1. f1:**
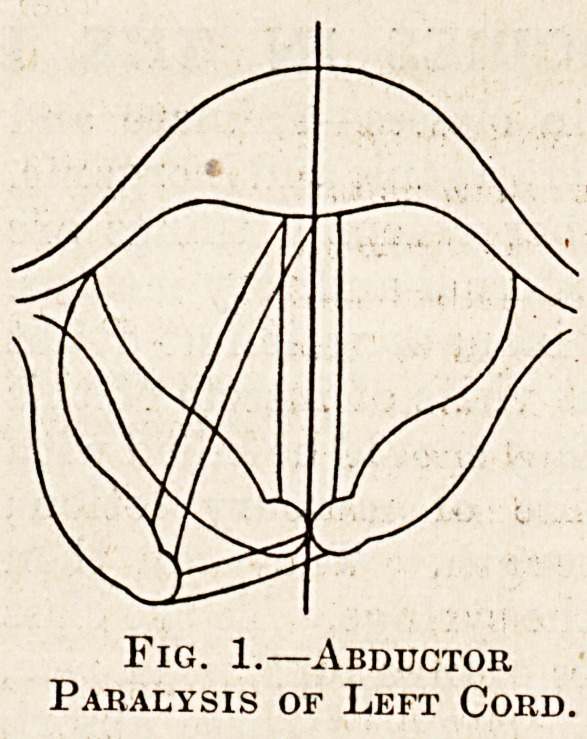


**Fig. 2. f2:**